# Long non-coding RNA ANRIL promotes homologous recombination-mediated DNA repair by maintaining ATR protein stability to enhance cancer resistance

**DOI:** 10.1186/s12943-021-01382-y

**Published:** 2021-07-05

**Authors:** Lei Liu, Yuanyuan Chen, Yijuan Huang, Kun Cao, Tingting Liu, Hui Shen, Jianguo Cui, Bailong Li, Jianming Cai, Fu Gao, Yanyong Yang

**Affiliations:** 1 Department of Radiation Medicine, Faculty of Naval Medicine, Naval Medical University, 800, Xiangyin Road, Shanghai, 200433 P. R. China; 2 Department of Oncology, General Hospital of Central Theater Command of Chinese People’s Liberation Army, No.627 Wuluo Road, Wuchang District, Wuhan, Hubei 430070 P. R. China; 3 School of Public Health and Management, Wenzhou Medical University, University Town, Wenzhou, Zhejiang P. R. China

**Keywords:** Long non-coding RNA, Cancer resistance, DNA damage repair, ATR

## Highlights

1. ANRIL promoted HR repair and conferred cancer resistance to DNA damage treatments
2. ANRIL bind with the ATR protein to maintain protein stability and protect against ubiquitination-mediated degradation
3. Loss of ANRIL disabled HR repair and increases radiosensitivity in lung cancer xenografts

## Main text

Aberrantly enhanced DNA damage repair leads to therapeutic resistance in many types of cancer [1, 2]. Recently, long non-coding RNAs (lncRNAs) have been shown as indispensable participants in DNA damage repair and provide novel targets for overcoming cancer resistance [3, 4]. However, the exact roles of most lncRNAs in DNA damage repair remain largely unknown. Here, we present our novel finding that lncRNA ANRIL promotes cancer resistance by mediating homologous recombination (HR) repair of DNA damage.

### ANRIL promoted HR repair to enhance cancer resistance to DNA damage treatments in lung cancer cells

**Fig. 1:**
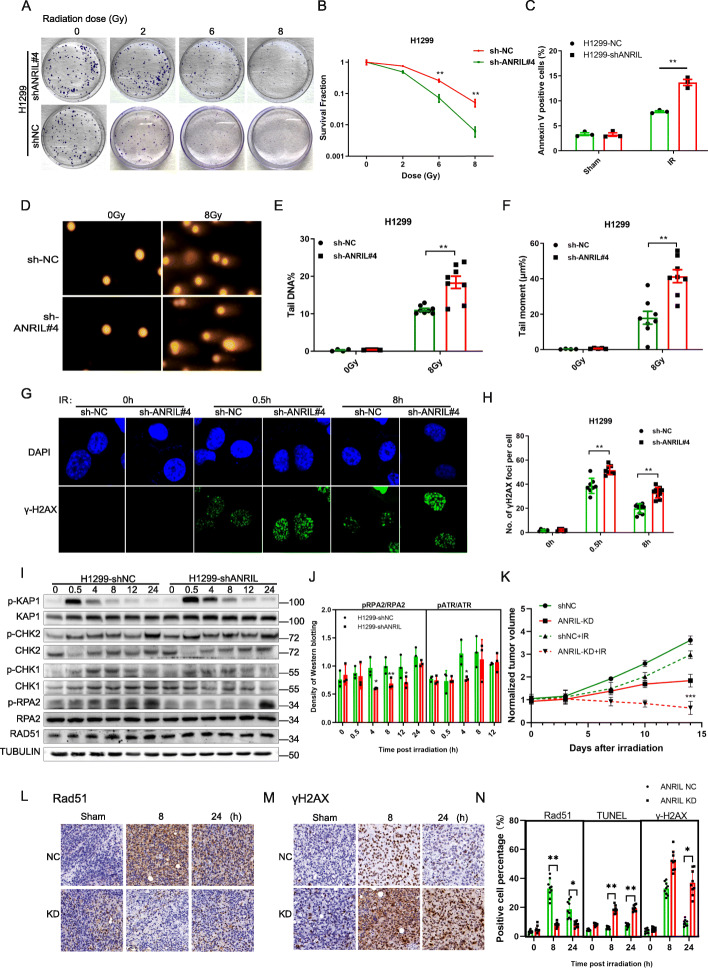
ANRIL promoted HR repair to enhance cancer resistance in lung cancer cells. A: Representative images of the clonogenic survival assay of ANRIL NC or ANRIL-knockdown H1299 cells after 0, 2, 4, 8 Gy irradiation. B: Quantitative analysis of the clonogenic survival assay results of ANRIL-KD H1299 cells that received the indicated IR treatment. Cells transfected with shNC served as controls. Error bars represent the SEM of the mean of 3 independent experiments, two tailed Student’s *t* test. **P < 0.01. C: Cell apoptosis was measured with flow cytometry via the Annexin V and PI double staining method in ANRIL NC and ANRIL-KD cells at 24 h after 8 Gy irradiation. Error bars represent the SEM of the mean of 3 independent experiments, two tailed Student’s *t* test. **P < 0.01. D-F: Representative images from the comet assay of ANRIL-knockdown or control cells at 8 h after 8 Gy irradiation. The tail DNA percentage (E) and tail moment (F) were quantified from comet assay images in ANRIL-KD or NC cells. Error bars represent the SEM of the mean of 3 independent experiments, two tailed Student’s *t* test. **P < 0.01. G, H: Images and quantitative results of the γH2AX foci assay of NC and ANRIL-KD cells at the indicated time points after 8 Gy irradiation. Error bars represent the SEM of the mean of 3 independent experiments, two tailed Student’s *t* test. **P < 0.01. I: Representative images and of Western blotting of RPA2 phosphorylation, Rad51 phosphorylation, Chk1 phosphorylation, Chk2 phosphorylation and Kap1 phosphorylation in ANRIL-knockdown cells after irradiation. J: quantitative analysis of RPA2 phosphorylation and ATR phosphorylation in ANRIL-NC and ANRIL-KD cells. ANRIL NC cells were used as controls. The data are shown as the mean ± SEM. Significance was determined with Student’s t test. **P < 0.01, *P < 0.05. K: volumes grow curves of tumors isolated from the NC and ANRIL-KD groups with/without irradiation. Data are shown as the mean ± SD, *n* = 9, two-tailed Student’s *t* test. ***P < 0.001. L-M: Representative images of immunochemically stained Rad51 (L) and γH2AX (M) in ANRIL NC and-KD tumors at 0, 8, and 24 h after local irradiation (*n =* 9). N: Quantitative analysis of the percentages of RAD51-, TUNEL- and γH2AX-positive cells from IHC images from the indicated groups. Data are shown as the mean ± SD, *n =* 9, two-tailed Student’s *t* test. **P < 0.01, *P < 0.05

Lung cancer with high ANRIL expression is a leading cause of mortality worldwide and was chosen as a model to investigate the role of ANRIL in DNA damage repair and therapeutic resistance [5]. First, significant elevation of ANRIL was found in lung cancer tissues compared with that in adjacent normal tissues from 80 pairs of clinical specimens (Fig. S1A). High expression of ANRIL was also confirmed in lung cancer cells compared with that in normal BEAS-2B cells (Fig. S1B). Through analyzing data from TCGA database, ANRIL was also found to be upregulated in lung squamous cell carcinoma, but not adenocarcinoma (Fig. S1K). Moreover, ANRIL expression was greatly increased by ionizing radiation (IR) and other DNA damaging reagents, such as etoposide and camptothecin (CPT) (Fig. S1C-F), which was supported by the results of a previous study [6]. Then, genetically modified H1299 cells (ANRIL^high^) or H460 cells (ANRIL^low^) containing ANRIL-knockdown or ANRIL-overexpression plasmids, respectively, were used to investigate the role of ANRIL in cancer resistance (Fig. S1G, H). Compared with those in the control group, ANRIL knockdown in H1299 cells significantly reduced the colony-forming efficiency and increased cell apoptosis when combined with IR treatment (Fig. 1A-C). In contrast, overexpression of ANRIL in H460 cells significantly increased colony formation and inhibited radiation-induced cell apoptosis (Fig. S2A-C). These data indicate that the increased ANRIL expression confers resistance to ionizing radiation in lung cancer cells.

Then, we investigated whether ANRIL played a critical role in DNA damage repair. In the neutral comet assay, more DNA damage was observed at 8 h in irradiated ANRIL-KD cells than that in NC cells (Fig. 1D-F). In contrast, less DNA damage was observed in ANRIL-OE H460 cells after irradiation (Fig. S2D-F). These results suggested that loss of ANRIL resulted in unrepaired DNA damage after IR, which prompted us to monitor the DNA repair kinetics. Through in situ phos-Histone 2AX (γH2AX) and 53BP1 foci assays, we found that more γH2AX foci remained unresolved at 8 h after irradiation than those in control cells (Fig. 1G, H), while the decreased numbers of γH2AX foci per nucleus were observed in ANRIL-OE H460 cells (Fig. S2G, H). Moreover, after the number of foci was normalized to that at 0.5 h, a significant difference was found at 8 h after IR, indicating dysfunction of DNA damage repair when ANRIL was knockdown (Fig. S2I).

**Fig. 2:**
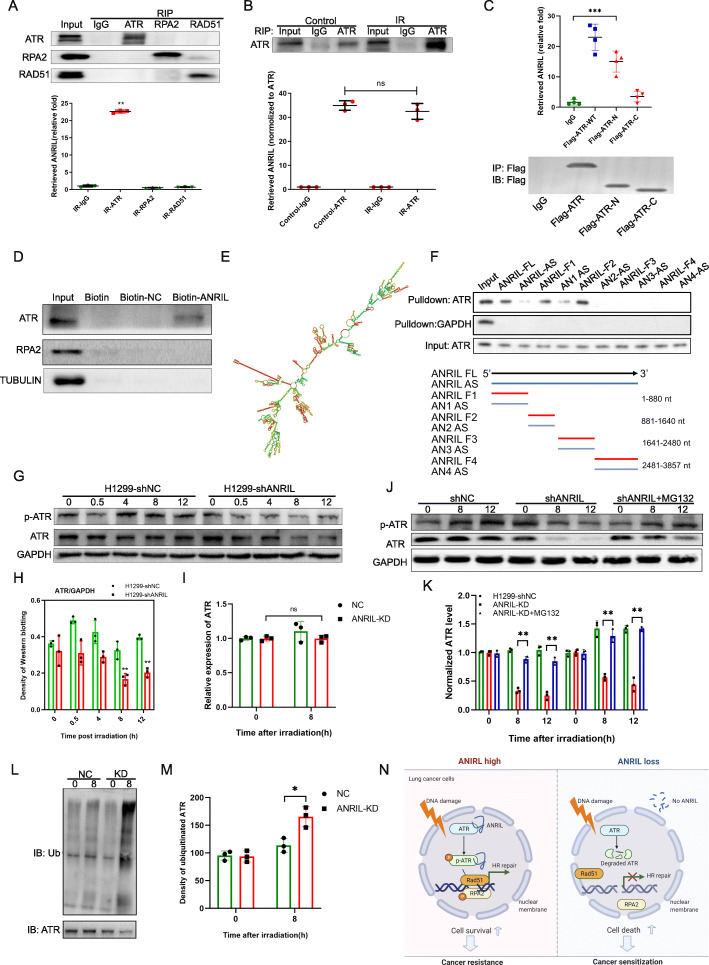
ANRIL directly binds with ATR to maintain the stability of the ATR protein. A: upper, Representative images of RNA immunoprecipitation (RIP) with antibodies against ATR, RPA2 and RAD51; lower: RIP-qPCR assay of the relative expression of ANRIL in ATR-, RPA2- and RAD51-precipitated extracts. Error bars represent the SD of the mean of *n* = 3 experiments. RIP with IgG was used as a negative control. **P < 0.01 versus the IgG group as determined by two-tailed Student’s *t* test. B: RIP-qPCR assay of ANRIL expression in the presence of ATR antibodies with/without irradiation. The error bars represent the SD of the mean of *n =* 3 experiments. ns: non significance between control and IR group when normalized to ATR protein level. C: RIP-qPCR assay of ANRIL expression in the presence of Flag primary antibody in cells transfected with Flag-ATR, Flag-ATR-N and Flag-ATR-C. RIP with IgG was used as a negative control. ***P < 0.001 versus the IgG group as determined by two-tailed Student’s *t* test. D: Immunoblot assay of ATR, RPA2 and tubulin in the RNA pulldown extract with biotin-labeled full-length ANRIL. Biotin and Biotin-NC sequences were used as negative controls. E: Predicted structure of the lncRNA ANRIL determined by RNA fold software. F: Immunoblot of ATR in RNA pulldown extracts with different ANRIL fragments and their antisense (AS) sequences (1–880, 881–1640, 1641–2480, 2481–3857). G, H: Representative images (F) and quantitative analysis (G) of the Western blotting results of the ATR protein in H1299-NC and ANRIL-knockdown cells after 0, 4, 8, and 12 Gy irradiation. Phosphorylated ATR was also detected. The data are shown as the mean ± SEM. Significance was determined with Student’s *t* test. **P < 0.01. I: Real-time PCR assay of ATR mRNA expression in ANRIL NC and ANRIL-KD cells after irradiation. The data are shown as the mean ± SEM. NS, non-significance was observed with Student’s *t* test. J: Western blot analysis of pATR and ATR protein in ANRIL-knockdown cells pretreated with the proteasome inhibitor MG132. NC was used as positive control. K: Quantitative analysis was performed with ImageJ software. The data are shown as the mean ± SEM. Significance was determined with Student’s *t* test. **P < 0.01. L: Immunoprecipitation analysis of ubiquitinated ATR-irradiated ANRIL NC and ANRIL-KD cells. M: Quantitative analysis of ubiquitinated ATR was performed with ImageJ software. Error bars represent the SD of the mean of *n* = 3 experiments, *P < 0.05. N: Schematic diagram of how the lncRNA ANRIL regulates HR repair and radiosensitivity

After knowing the necessary of ANRIL during DNA damage repair, the exact functions of ANRIL in the DNA damage response (DDR) were further investigated continuously in this study. First, phosphorylation of ATM and ATR after irradiation was attenuated in ANRIL-KD H1299 cells (Fig. S3C, Fig. 2G). Then, we determined the downstream factors of ATR signaling pathway and found that activation of RPA2 and CHK1 was also abrogated in ANRIL-KD H1299 cells (Fig. 1I, J). In contrast, ANRIL OE increased the phosphorylation of ATR and Chk1 (Fig. S3D). No obvious difference was observed in the phosphorylation of CHK2 or KAP1, which are substrates of ATM [7]. These results indicate that ANRIL may be critical for the ATR-Chk1/RPA2 axis. As expected, both the number of ATR foci and RPA2 foci per cell were significantly reduced in ANRIL-KD cells (Fig. S3E). The ATR-CHK1 and RPA2 signaling pathways are critical for cell cycle arrest and HR repair, suggesting that ANRIL is critical for effective HR repair of DNA damage [8, 9]. In addition, we performed RNA sequencing of irradiated ANRIL-proficient and ANRIL-deficient cells and found that DNA damage response-related signaling pathways, including the p53 signaling pathway [10], were also affected (Fig. S3A, B). The above results show that ANRIL is required for DDR and increasing cancer resistance in lung cancer cells.

To explore the influence of ANRIL on cancer resistance in vivo, NC and ANRIL-knockdown H1299 cells as well as vector- and ANRIL-overexpressing H460 cells were injected subcutaneously into nude mice (Fig. S5A). ANRIL knockdown combined with local irradiation resulted in reduced tumor growth in terms of tumor volume curve and weight (Fig. 1K, S5B). Consistently, ANRIL-OE H460 cells showed increased tumor resistance to radiotherapy compared with the vector group (Fig. S7A-D). These in vivo experimental results confirmed the role of ANRIL in radiotherapy resistance. Among the key factors in HR repair, the protein levels of Rad51 and RPA2 in tumor tissues were also reduced (Fig. 1L, N, S6E, F). Tumors derived from ANRIL-KD cells showed more unrepaired DNA damage (γH2AX) and more cell apoptosis (TUNEL) after irradiation (Fig. 1M, N). Fewer proliferating cells (Ki67 staining) were observed in tumors derived from ANRIL-KD cells (Fig. 1N; S6C, D; S5D). Targeting HR repair is an important strategy to sensitize tumors to radiotherapy, which also increases the efficacy of PARP inhibitors [11, 12]. Our findings provide a novel target to increase the effectiveness of cancer therapy by abrogating ANRIL mediated HR repair.

### ANRIL directly binds with ATR to maintain stability of the ATR protein and protect against ubiquitination-mediated degradation

The inactivation of ATR and HR repair in ANRIL-deficient cells prompted us to investigate the underlying mechanisms of these effects. ANRIL was found to be located in the nucleus through the RNA FISH assay (Fig. S2J), which suggested it may play a role in directly regulating DNA damage repair. In order to identify whether ANRIL interacts with its direct targets during HR repair, RIP experiments were performed by using ATR-, RAD51- and RPA2-specific antibodies. Surprisingly, ANRIL was enriched in the protein-RNA complex immunoprecipitated by an ATR-specific antibody instead of a RPA2 or RAD51 antibody (Fig. 2A), suggesting that ANRIL may play a role in regulating ATR function. In irradiated H1299 cells, more ATR-bound ANRIL was observed; however, no significant difference was found when ATR-bound ANRIL was normalized to the ATR protein, which suggested that the ATR protein instead of ATR-ANRIL binding was affected by IR (Fig. 2B). After knowing the results of RIP experiments with Flag primary antibody, the cells were transfected with Flag-ATR, Flag-ATR-N and Flag-ATR-C plasmid in order to map the region of ATR interacting with ANRIL. The data showed that the N terminal of ATR mainly accounted for its interaction with ANRIL (Fig. 2C). Further investigations revealed that the ATR binding with ANRIL did not depend on its phosphorylation level (Fig. S3G). Moreover, the direct binding of ANRIL with the ATR protein was confirmed with an RNA pulldown assay (Fig. 2D). One of the most important functions of lncRNA is to bind to proteins, which leads to functional changes [13]; moreover, any ATR-binding lncRNA has not yet been reported.

To determine the detailed sequence of ANRIL that interacts with ATR, different fragments of functional regions of ANRIL were generated based on its secondary structure predicted with RNA fold software (Fig. 2E, F). Through an RNA pulldown assay, 5′-regions (0–880 bp and 881–1640 bp) were identified to be essential for binding with the ATR protein (Fig. 2F). These results demonstrate that ANRIL directly binds to ATR at the N-terminus, the significance of which needs to be investigated in future studies.

Dysfunction of ATR often results in defects in repairing DNA damage and replication stress [14], which prompted us to investigate the significance of ANRIL-ATR binding. We surprisingly observed that the protein level of ATR declined in ANRIL-KD H1299 cells but not in normal H1299 cells when DNA damage treatment was applied (Fig. 2G, H), but remained unchanged in control cells (Fig. S3F). Consistently, the level of ATR decreased dramatically in ANRIL-KD tumors but not in tumors derived from NC cells (Fig. S6A, B). However, the mRNA level of ATR remained unchanged (Fig. 2I), suggesting possible post-translational regulation of the ATR protein. Ubiquitination is an important type of protein degradation and can be blocked by the proteasome inhibitor MG132 [15]. As expected, the ATR protein was retained in MG132-treated ANRIL-KD cells (Fig. 2J, K). Furthermore, in ANRIL-KD cells, ubiquitinated bands of the protein complex immunoprecipitated with an ATR-specific antibody were observed in irradiated cells (Fig. 2L, M).

As the key kinase in DDR, ATR may account for the critical role that ANRIL plays in DNA damage repair. We changed the expression of ATR by performing rescue experiments through overexpressing ATR in ANRIL-KD cells, and performing ATR knockdown in ANRIL-OE cells (Fig. S4A, E). Our data showed that ATR overexpression in ANRIL-KD cells significantly increased the efficacy of DNA repair and cell survival (Fig. S4B-D). However, ATR knockdown in ANRIL-OE cells inhibited DNA repair and cell survival (Fig. S4F-H). These results suggest that ANRIL directly binds to and protects ATR from ubiquitination-mediated degradation, and then, ANRIL promotes HR repair and cancer resistance.

In summary, our present study also provided the potential novel target in the investigation through disrupting ANRIL-ATR complex to conquer lung cancer. As indicated in NCCN guideline 2021, radiotherapy and adjuvant chemo-radiotherapy represent important strategies in the treatments of NSCLC [16]. However, cancer cells usually become to be concomitantly resistant along radiotherapy. Uncovering the underlying mechanism of this phenomenon is essential to overcome the radio-resistance and is of great importance to improve radiotherapy of lung cancer. DSB repair is vital to the outcome of both radio- and chemo- therapies, of which HR repair capacity can contribute to cancer resistance to many treatment reagents, including ionizing radiation, cisplatin, and PARP inhibitors [17, 18]. The ATR inhibitor berzosertib was also developed as novel treatment for overcoming platinum resistant lung cancer [19]. Targeting ATR also improves therapeutic index in preclinical lung cancer model [20]. Our present findings suggest that targeting ANRIL will lead to degradation of ATR, which will further result in defects of HR repair. Thus, these findings can provide the clues to realize the novel mechanism and obtain potential therapeutic applications, not only for radiotherapy but also for chemotherapies relative to ATR related HR repair.

## Conclusion

Our study identified an important ATR-interacting lncRNA, ANRIL, and uncovered its direct mechanism in HR repair of DNA damage: maintaining the stability of the ATR protein. Loss of ANRIL led to degradation of the ATR protein via ubiquitination. In addition to our mechanistic findings, we also demonstrated that ANRIL is a novel target for overcoming cancer resistance to ionizing radiation. ANRIL depletion resulted in inhibition of tumor growth and an increase in tumor radiosensitivity, suggesting that the lncRNA ANRIL is a potential therapeutic target for lung cancer. (Fig. 2N).

## Supplementary Information

**Additional file 1 **: **Supplementary Fig. 1**: A: Relative expression levels of ANRIL in lung cancer tissues and adjacent normal tissues. *N* = 80, Significance was determined with Student’s *t* test. ***P < 0.001. B: Relative expression levels of ANRIL in lung cancer cell lines, including A549, H460, H1299, and H1975, and the normal cell line BEAS-2B. The data are shown as the mean ± SEM, *n* = 3 independent experiments, and significance was determined with Student’s *t* test. *P < 0.05, **P < 0.01, ***P < 0.001 versus BEAS-2B cells. C: Relative expression of ANRIL at different time points after 8 Gy irradiation or at 12 h after different doses of irradiation (D). The data are shown as the mean ± SEM, *n* = 3 independent experiments, and significance was determined with Student’s *t* test. *P < 0.05, **P < 0.01, ***P < 0.001 versus unirradiated cells. E, F: Relative expression of ANRIL in H1299 cells at different time points after release by treatment with etoposide (100 mg mL, 4 h) or CPT (1 μM, 1 h). The data are shown as the mean ± SEM, *n =* 3 independent experiments, and significance was determined with Student’s *t* test. *P < 0.05, **P < 0.01, versus untreated cells. G, H: Relative expression level of ANRIL in H1299 cells transfected with the NC or shANRIL vector (G) and in H460 cells transfected with the ANRIL overexpression vector (H). The data are shown as the mean ± SD, *n =* 3 independent experiments, two-tailed Student’s *t* test. **P < 0.001, ***P < 0.001. I: Relative expression of ANRIL in Ku55933, NU7441, VE821 pretreated cells after irradiation. *P < 0.05. J: Relative expression of ANRIL in H1299 cells pretreated with Actinomycin D for 1 h. K: ANRIL expression in lung cancer derived from TCGA database. *P <* 0.05.

**Additional file 2 **: **Supplementary Fig. 2:** A: Representative images of the clonogenic survival assay of the vector or ANRIL-OE H460 cells after 0, 2, 4, and 8 Gy irradiation. B: Quantitative analysis of the clonogenic survival assay of control and ANRIL-OE cells with the indicated IR treatment. Cells transfected with the vector served as controls. Error bars represent the SEM of the mean of 3 independent experiments, two tailed Student’s *t* test. **P < 0.01. C: Apoptotic cells (Annexin V positive) were measured with flow cytometry in the vector and ANRIL-OE cells at 24 h after 8 Gy irradiation. Error bars represent the SEM of the mean of 3 independent experiments, two tailed Student’s *t* test. **P < 0.01. D: Representative images of the comet assay of ANRIL-OE (D) or control cells at 8 h after 8 Gy irradiation. Tail DNA percentage (E) and tail moment (F) were quantified from comet assay images of ANRIL-OE or control cells. Error bars represent the SEM of the mean of 3 independent experiments, two tailed Student’s *t* test. **P < 0.01. G, H: Representative images (G) and quantitative foci number (H, bar = 20 nm) of the γH2AX staining assay of the vector and ANRIL-overexpressing cells. Error bars represent the SEM of the mean of 3 independent experiments, two tailed Student’s *t* test. **P < 0.01. I: The number of γH2AX foci per cell were normalized to the foci at 0.5 h. Error bars represent the SEM of the mean of 3 independent experiments, two tailed Student’s *t* test. *P < 0.05. J: Representative images of the RNA FISH assay to determine the subcellular localization of ANRIL.

**Additional file 3 **: **Supplementary Fig. 3:** A: Heatmap of differentially expressed genes involved in the p53 signaling pathway, PARP signaling pathway and PI3K-Akt pathway in ANRIL NC cells and ANRIL-KD cells. B: The top 30 signaling pathways enriched with differentially expressed genes from ANRIL NC and ANRIL-KD cells according to the RNA sequencing results. C: Western blot analysis of the phosphorylation of ATR, ATM, p53, and p21 in H1299 and ANRIL-knockdown cells after irradiation. D: Western blot analysis of ATR, RPA2, Chk1, p21, Kap1 and Chk2 phosphorylation in ANRIL-OE cells after irradiation. E: Representative images and quantitative analysis of RPA2 foci and ATR foci in irradiated ANRIL-KD and normal cells. Quantitative analysis of the RPA2 foci number per nucleus in different groups. The data are shown as the mean ± SEM, *n* = 3 independent experiments, and significance was determined with Student’s *t* test. *P < 0.05, **P < 0.01. F: Representative image of Western blotting of ATR at 24, 48, and 72 h after shANRIL transfection. G: RIP-qPCR assay of ANRIL expression in the presence of pATR and ATR primary antibody in irradiated H1299 cells. RIP with IgG was used as a negative control. NS versus the IgG group as determined by two-tailed Student’s *t* test.

**Additional file 4 **: **Supplementary Fig. 4:** A: The ATR protein was analyzed via Western blot analysis in NC, ANRIL-KD- and pcDNA 3.1-, and pcDNA 3.1-ATR-transfected cells. GAPDH was used as an internal control. B: Quantitative analysis of the clonogenic survival assay of ANRIL-KD and ATR-OE cells that received the indicated IR treatment. Error bars represent the SEM of the mean of 3 independent experiments, two tailed Student’s *t* test. **P < 0.01. C, D: Images and quantitative results of the γH2AX staining assay of NC and ANRIL-KD cells at the indicated time points after 8 Gy irradiation. Error bars represent the SEM of the mean of 3 independent experiments, two tailed Student’s *t* test. ***P < 0.001. E: Western blotting analysis of ATR in ANRIL-OE-, siNC- and siATR-transfected cells. F: Quantitative analysis of the clonogenic survival assay of ANRIL-OE and ATR-KD cells with the indicated IR treatment. Error bars represent the SEM of the mean of 3 independent experiments, two tailed Student’s *t* test. *P < 0.05. G, H: Images and quantitative results of the γH2AX staining assay of ANRIL-OE and ATR-KD cells after 8 Gy irradiation. Error bars represent the SEM of the mean of 3 independent experiments, two tailed Student’s *t* test. *P < 0.05.

**Additional file 5 **: **Supplementary Fig. 5:** A: A flow chart to illustrate the overall design of the animal study. B: The weight of tumors isolated from the NC and ANRIL-KD groups with/without irradiation at 14 days after irradiation. Data are shown as the mean ± SD, *n* = 9, two-tailed Student’s *t* test. ***P < 0.001. C: Representative images of tumor-bearing mice arising from ANRIL NC or ANRIL-KD cells with/without 10 Gy local irradiation. D: Representative images of TUNEL immunochemically stained tissue sections from tumors from ANRIL-KD and NC lung cancer tissues. E: Images of tumors isolated from four groups: NC, NC + IR, ANRIL-KD, ANRIL-KD + IR (*n* = 9).

**Additional file 6 **: **Supplementary Fig. 6:** IHC staining and quantification of the ATR protein (A, B), Ki67 (C, D) and RPA2 (E, F) in irradiated tumor tissues derived from ANRIL NC and ANRIL-KD H1299 cells. The positive percentages of ATR, Ki67 and RPA2 were measured with ImageJ software. The data are shown as the mean ± SEM. Significance was determined with Student’s *t* test (*n =* 9). *P < 0.05, **P < 0.01.

**Additional file 7 **: **Supplementary Fig. 7:** A: Representative images of tumor-bearing mice with/without 10 Gy irradiation. B: Representative images of tumors isolated from four different groups: NC, NC + IR, ANRIL-OE, ARNIL-OE + IR. C: The volume growth curves were monitored every four days after local irradiation. Error bars represent the SD of in vivo experiments (*n =* 9). *P < 0.05, **P < 0.01 versus the control group. D: The weight (g) of tumors isolated from the NC and ANRIL-OE groups with/without irradiation. Data are expressed as the mean ± SEM. Significance was determined with Student’s *t* test. *P < 0.05, **P < 0.01. E: Representative images of Ki67-stained tissue sections from tumors isolated from ANRIL-OE and NC lung cancer tissues. F: Quantitative analysis of the Ki67-positive cell percentage in different groups. Data are expressed as the mean ± SEM. Significance was determined with Student’s *t* test. *P < 0.05.

**Additional file 8.**

**Additional file 9.**

**Additional file 10.**

## Data Availability

The datasets used and analyzed during the current study are available within the manuscript and its additional files.

## Abbreviations

DSB: DNA double strand breaks
NHEJ: Non-homologous end joining
HR: Homologous recombination
ATR: Ataxia Telangiectasia and Rad3-Related Protein
lncRNA: long non-coding RNAs
ANRIL: Antisense non-coding RNA in the INK4locus
NSCLC: Non-small cell lung cancer
RIP: RNA immunoprecipitation
