# Supplementary figures and images for: Long non-coding RNA ANRIL promotes homologous recombination-mediated DNA repair by maintaining ATR protein stability to enhance cancer resistance
Source: Mol Cancer. 2021 Jul 5;20:94. doi: 10.1186/s12943-021-01382-y (PMC8256557; doi:10.1186/s12943-021-01382-y)

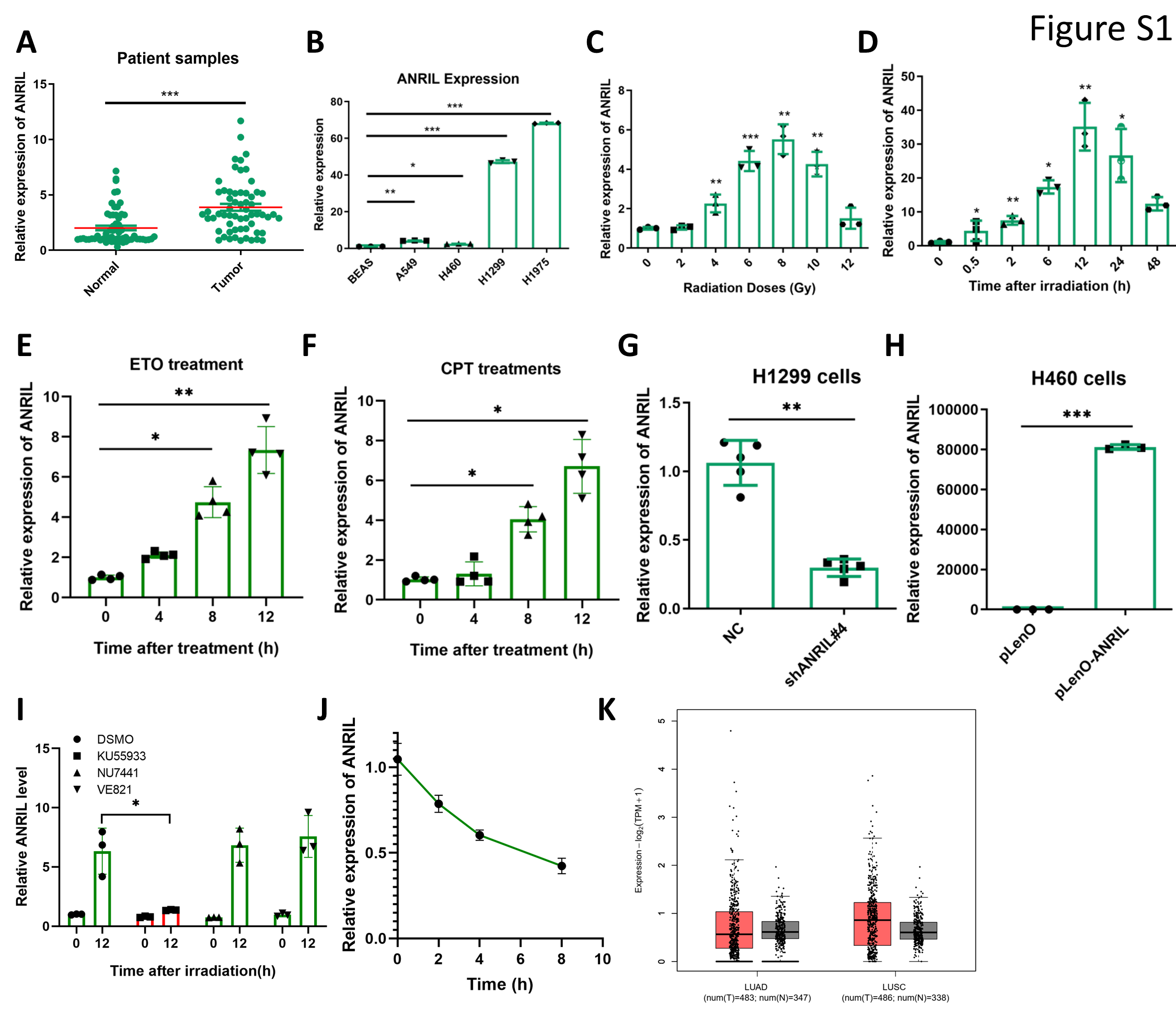

Supplement: Supplementary file 1 — Additional file 1 : Supplementary Fig. 1: A: Relative expression levels of ANRIL in lung cancer tissues and adjacent normal tissues. N = 80, Significance was determined with Student’s t test. ***P < 0.001. B: Relative expression levels of ANRIL in lung cancer cell lines, including A549, H460, H1299, and H1975, and the normal cell line BEAS-2B. The data are shown as the mean ± SEM, n = 3 independent experiments, and significance was determined with Student’s t test. *P < 0.05, **P < 0.01, ***P < 0.001 versus BEAS-2B cells. C: Relative expression of ANRIL at different time points after 8 Gy irradiation or at 12 h after different doses of irradiation (D). The data are shown as the mean ± SEM, n = 3 independent experiments, and significance was determined with Student’s t test. *P < 0.05, **P < 0.01, ***P < 0.001 versus unirradiated cells. E, F: Relative expression of ANRIL in H1299 cells at different time points after release by treatment with etoposide (100 mg mL, 4 h) or CPT (1 μM, 1 h). The data are shown as the mean ± SEM, n = 3 independent experiments, and significance was determined with Student’s t test. *P < 0.05, **P < 0.01, versus untreated cells. G, H: Relative expression level of ANRIL in H1299 cells transfected with the NC or shANRIL vector (G) and in H460 cells transfected with the ANRIL overexpression vector (H). The data are shown as the mean ± SD, n = 3 independent experiments, two-tailed Student’s t test. **P < 0.001, ***P < 0.001. I: Relative expression of ANRIL in Ku55933, NU7441, VE821 pretreated cells after irradiation. *P < 0.05. J: Relative expression of ANRIL in H1299 cells pretreated with Actinomycin D for 1 h. K: ANRIL expression in lung cancer derived from TCGA database. P < 0.05. [file 12943_2021_1382_MOESM1_ESM.tif]

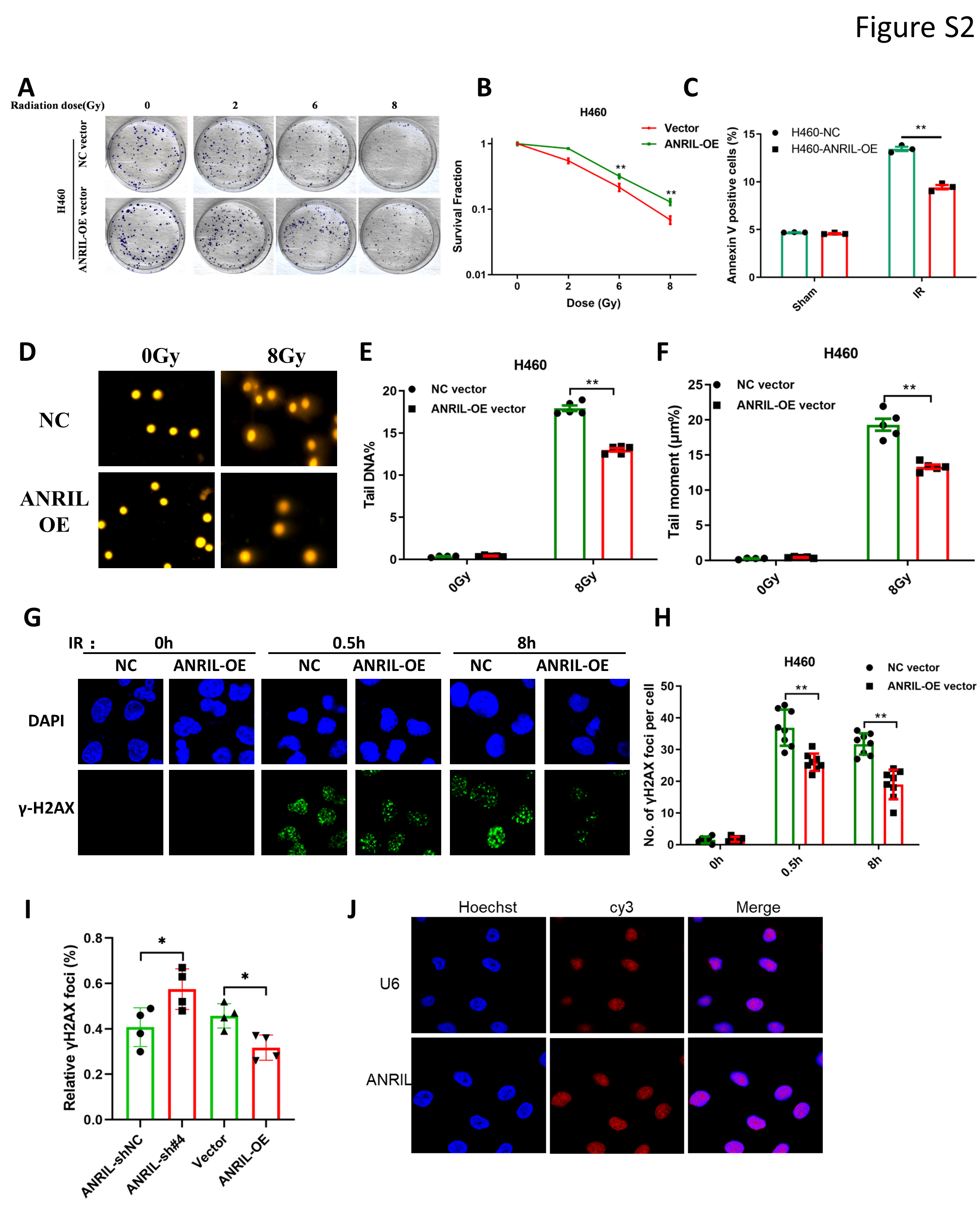

Supplement: Supplementary file 2 — Additional file 2 : Supplementary Fig. 2: A: Representative images of the clonogenic survival assay of the vector or ANRIL-OE H460 cells after 0, 2, 4, and 8 Gy irradiation. B: Quantitative analysis of the clonogenic survival assay of control and ANRIL-OE cells with the indicated IR treatment. Cells transfected with the vector served as controls. Error bars represent the SEM of the mean of 3 independent experiments, two tailed Student’s t test. **P < 0.01. C: Apoptotic cells (Annexin V positive) were measured with flow cytometry in the vector and ANRIL-OE cells at 24 h after 8 Gy irradiation. Error bars represent the SEM of the mean of 3 independent experiments, two tailed Student’s t test. **P < 0.01. D: Representative images of the comet assay of ANRIL-OE (D) or control cells at 8 h after 8 Gy irradiation. Tail DNA percentage (E) and tail moment (F) were quantified from comet assay images of ANRIL-OE or control cells. Error bars represent the SEM of the mean of 3 independent experiments, two tailed Student’s t test. **P < 0.01. G, H: Representative images (G) and quantitative foci number (H, bar = 20 nm) of the γH2AX staining assay of the vector and ANRIL-overexpressing cells. Error bars represent the SEM of the mean of 3 independent experiments, two tailed Student’s t test. **P < 0.01. I: The number of γH2AX foci per cell were normalized to the foci at 0.5 h. Error bars represent the SEM of the mean of 3 independent experiments, two tailed Student’s t test. *P < 0.05. J: Representative images of the RNA FISH assay to determine the subcellular localization of ANRIL. [file 12943_2021_1382_MOESM2_ESM.tif]

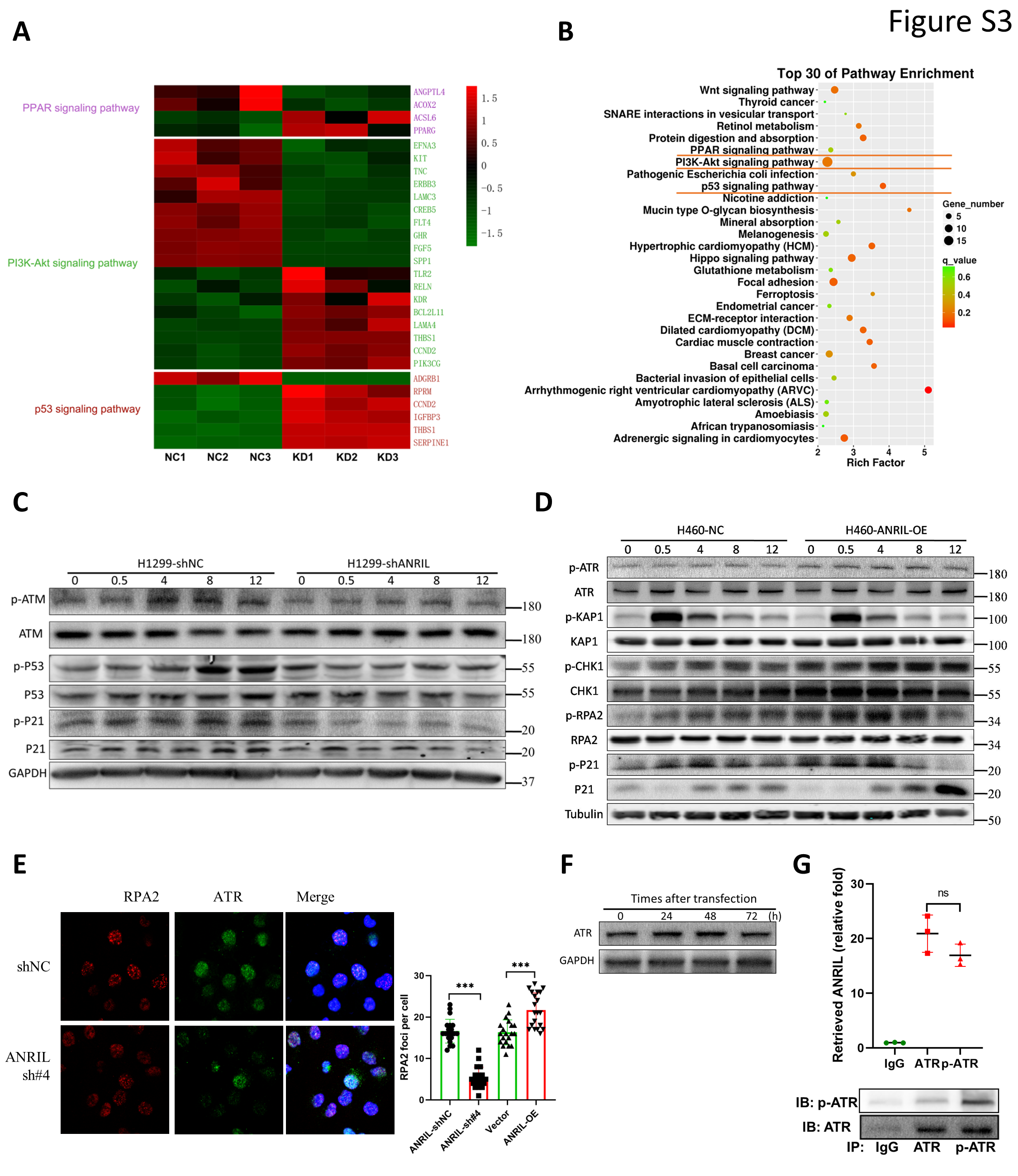

Supplement: Supplementary file 3 — Additional file 3 : Supplementary Fig. 3: A: Heatmap of differentially expressed genes involved in the p53 signaling pathway, PARP signaling pathway and PI3K-Akt pathway in ANRIL NC cells and ANRIL-KD cells. B: The top 30 signaling pathways enriched with differentially expressed genes from ANRIL NC and ANRIL-KD cells according to the RNA sequencing results. C: Western blot analysis of the phosphorylation of ATR, ATM, p53, and p21 in H1299 and ANRIL-knockdown cells after irradiation. D: Western blot analysis of ATR, RPA2, Chk1, p21, Kap1 and Chk2 phosphorylation in ANRIL-OE cells after irradiation. E: Representative images and quantitative analysis of RPA2 foci and ATR foci in irradiated ANRIL-KD and normal cells. Quantitative analysis of the RPA2 foci number per nucleus in different groups. The data are shown as the mean ± SEM, n = 3 independent experiments, and significance was determined with Student’s t test. *P < 0.05, **P < 0.01. F: Representative image of Western blotting of ATR at 24, 48, and 72 h after shANRIL transfection. G: RIP-qPCR assay of ANRIL expression in the presence of pATR and ATR primary antibody in irradiated H1299 cells. RIP with IgG was used as a negative control. NS versus the IgG group as determined by two-tailed Student’s t test. [file 12943_2021_1382_MOESM3_ESM.tif]

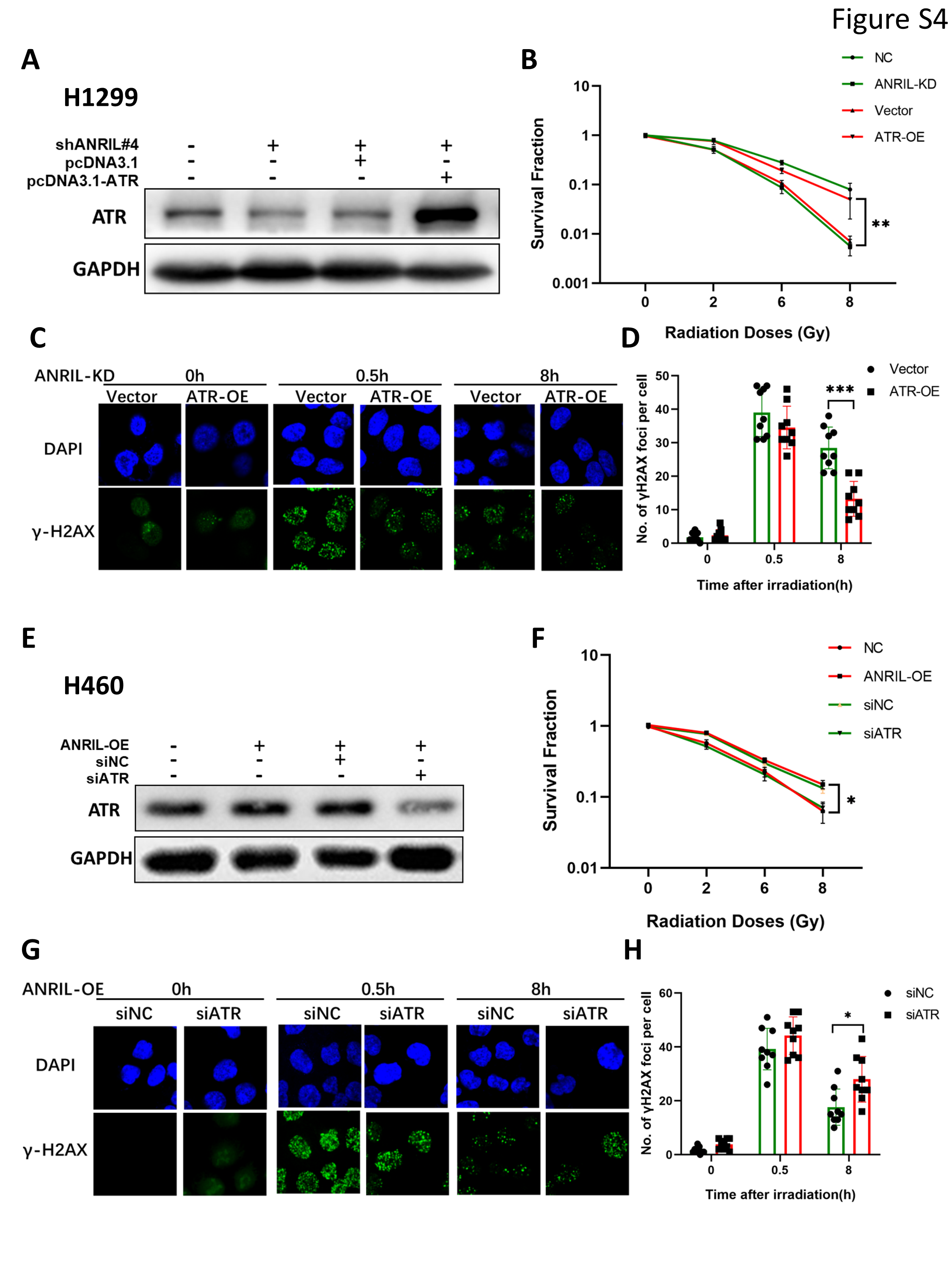

Supplement: Supplementary file 4 — Additional file 4 : Supplementary Fig. 4: A: The ATR protein was analyzed via Western blot analysis in NC, ANRIL-KD- and pcDNA 3.1-, and pcDNA 3.1-ATR-transfected cells. GAPDH was used as an internal control. B: Quantitative analysis of the clonogenic survival assay of ANRIL-KD and ATR-OE cells that received the indicated IR treatment. Error bars represent the SEM of the mean of 3 independent experiments, two tailed Student’s t test. **P < 0.01. C, D: Images and quantitative results of the γH2AX staining assay of NC and ANRIL-KD cells at the indicated time points after 8 Gy irradiation. Error bars represent the SEM of the mean of 3 independent experiments, two tailed Student’s t test. ***P < 0.001. E: Western blotting analysis of ATR in ANRIL-OE-, siNC- and siATR-transfected cells. F: Quantitative analysis of the clonogenic survival assay of ANRIL-OE and ATR-KD cells with the indicated IR treatment. Error bars represent the SEM of the mean of 3 independent experiments, two tailed Student’s t test. *P < 0.05. G, H: Images and quantitative results of the γH2AX staining assay of ANRIL-OE and ATR-KD cells after 8 Gy irradiation. Error bars represent the SEM of the mean of 3 independent experiments, two tailed Student’s t test. *P < 0.05. [file 12943_2021_1382_MOESM4_ESM.tif]

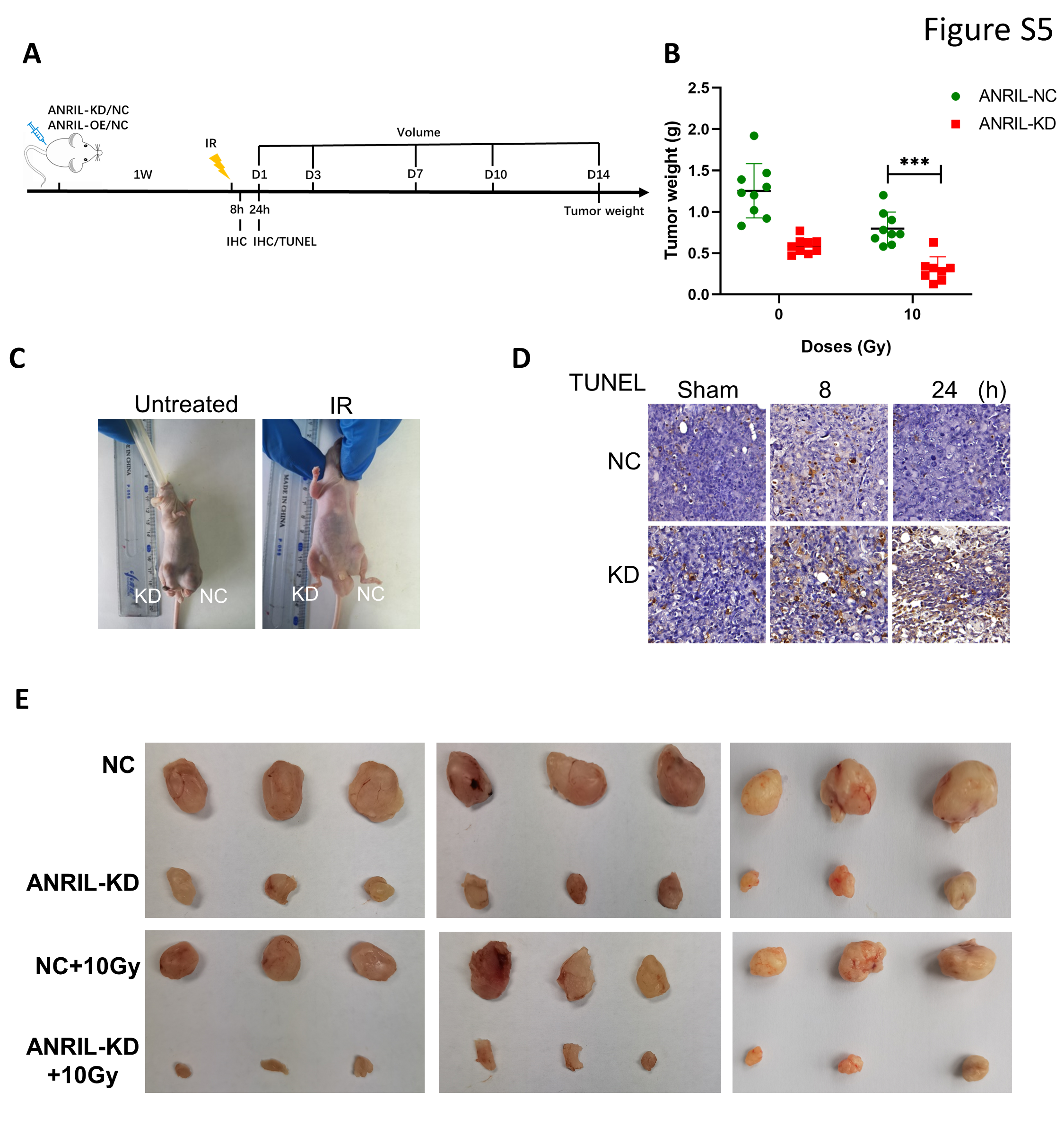

Supplement: Supplementary file 5 — Additional file 5 : Supplementary Fig. 5: A: A flow chart to illustrate the overall design of the animal study. B: The weight of tumors isolated from the NC and ANRIL-KD groups with/without irradiation at 14 days after irradiation. Data are shown as the mean ± SD, n = 9, two-tailed Student’s t test. ***P < 0.001. C: Representative images of tumor-bearing mice arising from ANRIL NC or ANRIL-KD cells with/without 10 Gy local irradiation. D: Representative images of TUNEL immunochemically stained tissue sections from tumors from ANRIL-KD and NC lung cancer tissues. E: Images of tumors isolated from four groups: NC, NC + IR, ANRIL-KD, ANRIL-KD + IR (n = 9). [file 12943_2021_1382_MOESM5_ESM.tif]

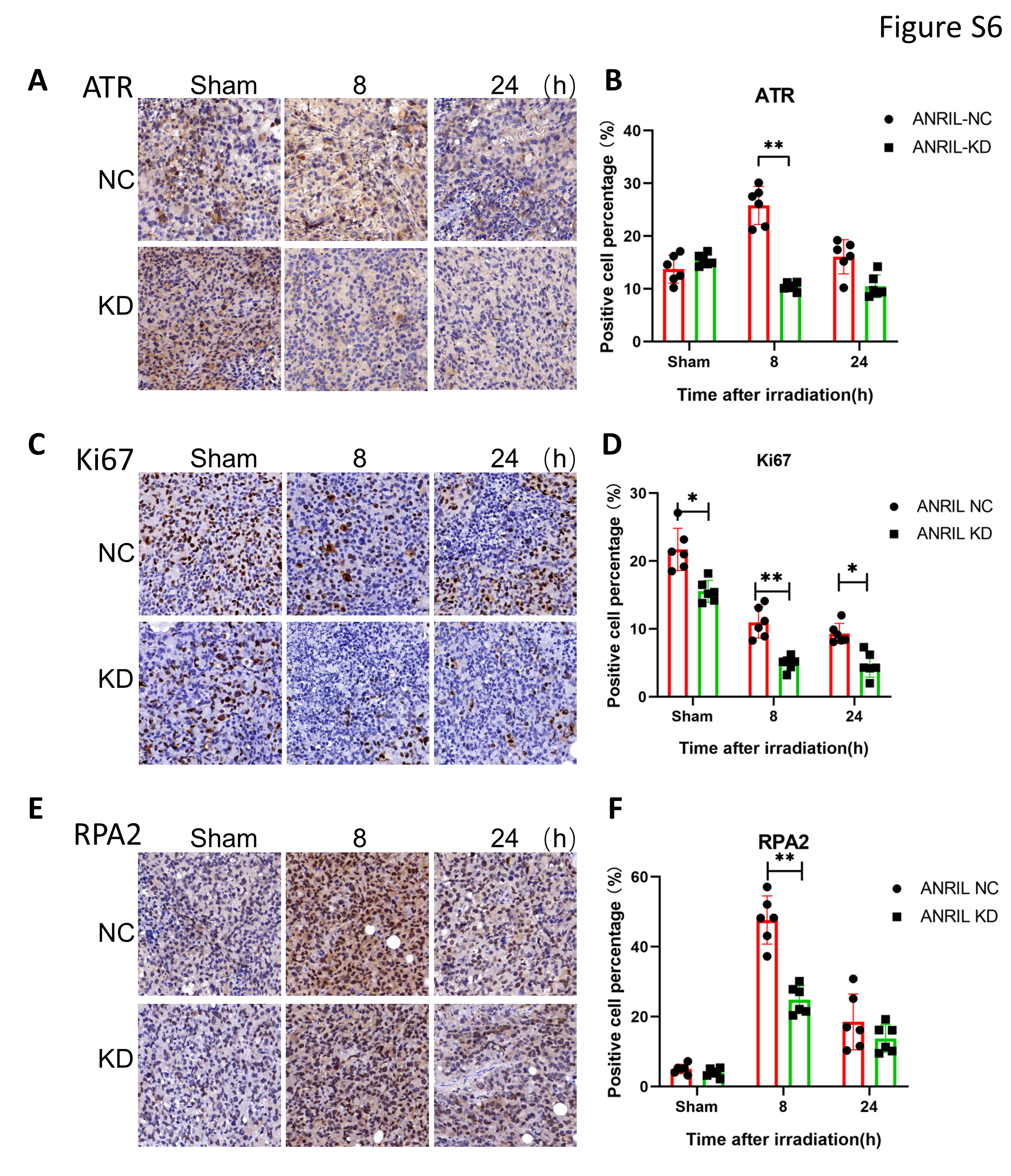

Supplement: Supplementary file 6 — Additional file 6 : Supplementary Fig. 6: IHC staining and quantification of the ATR protein (A, B), Ki67 (C, D) and RPA2 (E, F) in irradiated tumor tissues derived from ANRIL NC and ANRIL-KD H1299 cells. The positive percentages of ATR, Ki67 and RPA2 were measured with ImageJ software. The data are shown as the mean ± SEM. Significance was determined with Student’s t test (n = 9). *P < 0.05, **P < 0.01. [file 12943_2021_1382_MOESM6_ESM.tif]

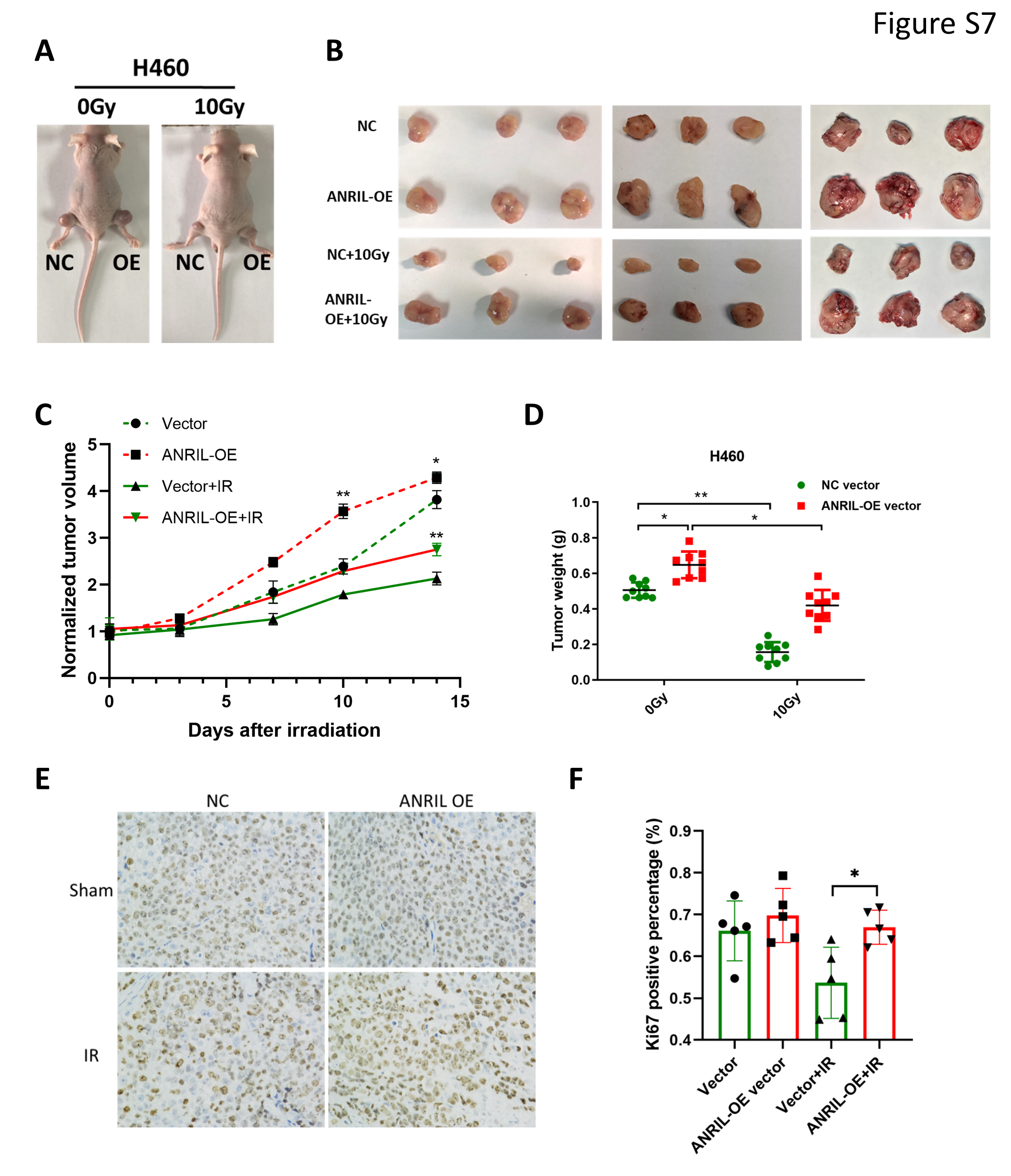

Supplement: Supplementary file 7 — Additional file 7 : Supplementary Fig. 7: A: Representative images of tumor-bearing mice with/without 10 Gy irradiation. B: Representative images of tumors isolated from four different groups: NC, NC + IR, ANRIL-OE, ARNIL-OE + IR. C: The volume growth curves were monitored every four days after local irradiation. Error bars represent the SD of in vivo experiments (n = 9). *P < 0.05, **P < 0.01 versus the control group. D: The weight (g) of tumors isolated from the NC and ANRIL-OE groups with/without irradiation. Data are expressed as the mean ± SEM. Significance was determined with Student’s t test. *P < 0.05, **P < 0.01. E: Representative images of Ki67-stained tissue sections from tumors isolated from ANRIL-OE and NC lung cancer tissues. F: Quantitative analysis of the Ki67-positive cell percentage in different groups. Data are expressed as the mean ± SEM. Significance was determined with Student’s t test. *P < 0.05. [file 12943_2021_1382_MOESM7_ESM.tif]
