# Supplementary material for: Long non-coding RNA ANRIL promotes homologous recombination-mediated DNA repair by maintaining ATR protein stability to enhance cancer resistance
Source: Mol Cancer. 2021 Jul 5;20:94. doi: 10.1186/s12943-021-01382-y (PMC8256557; doi:10.1186/s12943-021-01382-y)
Supplement: Supplementary file 8 — Additional file 8. [file 12943_2021_1382_MOESM8_ESM.docx]

**Materials and Methods**

**Cell lines and treatment**

Human non-small cell lung cancer H1299, H460, and A549 cells; human lung epithelial BEAS-2B cells; and human renal epithelial 293T cells were obtained from ATCC (American Type Culture Collection). H1299, H460, A549 and BEAS-2B cells were maintained in RPMI 1640 medium (HyClone) with 10% fetal bovine serum (Gibco, Scoresby, Victoria, Australia). The culture conditions of the 293T cells included DMEM with 10% FBS. In addition, 1% penicillin streptomycin-glutamine was added to the medium, and the cells were maintained at 37°C in a humidified incubator containing 5% CO_2_.

**Lentivirus packaging and stable cell line construction**

Logarithmic 293T cells were inoculated in a 10 cm dish at a cell density of 6-10×10^6^. According to the instructions of the viral packaging kit, ANRIL-knockdown (sh-ANRIL, Table S1) and ANRIL-overexpressing (ANRIL-OE, supplementary materials 2) target plasmids and viral plasmids were synthesized by Zuorun Biotech. Co. (Shanghai, China) and transfected into 293T cells. Then, the supernatants of the sh-ANRIL and ANRIL-OE cells were collected at 24 h, 48 h and 72 h after transfection. The collected virus supernatants were centrifuged at 3500 rpm/min for 10 minutes. Then, the virus supernatants were filtered through a filter of 0.45 μm, packaged and stored at -80°C. When the cell density of logarithmic H1299, H460 and A549 cells reached 60-70%, we added the ANRIL-knockdown virus supernatant to H1299 cells and the ANRIL-overexpressing virus supernatant to H460 and A549 cells. Polybrene (polyglutamine) was added to the culture medium for 48-72 hours. The constructed ANRIL-knockdown and ANRIL-overexpression target virus vectors were labeled with GFP to induce green fluorescence purinomycin resistance, and we observed the efficiency of infection under a fluorescence microscope. Appropriate amounts of purinomycin were added for screening, and the cells that survived for 72 hours were considered stable strains. The sequences of sh-ANRIL are listed in supplementary Table S1. For the ATR rescue experiments, the Flag-ATR plasmid or ATR siRNA (Sangon, China) was transfected into ANRIL-KD H1299 or ANRIL-OE H460 cells, respectively. At 48 h after transfection, the cells were irradiated, and then, the γH2AX foci assay was performed and the survival fraction was determined at the indicated time points.

**Patients and samples**

A total of 80 human nonsmall cell lung cancer (NSCLC) tissue specimens and corresponding adjacent normal tissue specimens were consecutively collected from patients undergoing surgical resection at the Department of Nuclear Radiation Shanghai Pulmonary Hospital, Tongji University (Shanghai, China), during the period from July 2018 to October 2018. None of the NSCLC patients received any treatments, including chemotherapy, radiotherapy, or any other medical intervention, prior to surgery and were diagnosed based on pathological evidence. The collected tissues were immediately snap-frozen in liquid nitrogen after resection and preserved in liquid nitrogen until RNA extraction. The study was compliant with all relevant ethical regulations for human research participants.

**Colony formation assay**

Cells in the logarithmic growth period were digested and placed in 60 mm dishes at the appropriate density. The irradiation dose of ^60^Co included 0 Gy, 2 Gy, 6 Gy and 8 Gy, with 3 replicates for each group. After irradiation, the cells were placed in an incubator to produce adherent cultures for 10-14 days. Then, the cells were fixed with 4% paraformaldehyde for 30 minutes at room temperature and stained with crystal violet staining solution (Beyotime, Shanghai, China) for 30 minutes after a gentle wash with PBS. Finally, the dishes were gently washed with running water and dried in air. Colonies consisting of >50 cells were counted under a microscope. The survival percentage was calculated as follows: (number of colonies/number of cells plated)/(number of colonies for corresponding control/number of cells plated).

**Apoptosis assay**

The Annexin V-APC/7-AAD Apoptosis Detection Kit was used to detect cell apoptosis according to the manufacturer’s instructions (Yeasen, Shanghai, China). Briefly, we collected the cell culture supernatant 24 hours after irradiation. After digesting the cells with trypsin (without EDTA), the cells were centrifuged. The supernatant was collected and washed three times with PBS. Then, we resuspended the cells in binding buffer at a concentration of approximately 5×10^5^ cells/mL and added Annexin V staining solution and 7-AAD according to the manufacturer’s instructions. The cells were incubated at room temperature for 10-15 minutes in the dark. Then, the cells were subjected to flow cytometry analysis (Beckman, USA).

**RNA isolation and Real-time PCR**

Total RNA was extracted from cells and tissues by using a Total RNA extraction kit (Tiangen, China) according to the manufacturer’s instructions. After the quality and quantity of the extracted RNA were confirmed by a nucleic acid quantitative detector (GeneQuant Pro, USA), complementary DNA (cDNA) was synthesized using PrimeScript^TM^ RT Master Mix (Perfect Real Time) (TaKaRa, Japan) according to the manufacturer’s instructions. The TB Green^TM^ Premix Ex Taq^TM^ Kit (TaKaRa, Kusatsu, Shiga, Japan) was used for real-time PCR analysis on a PCR platform (Roche LightCycler 2.0, Germany) to determine the expression level of ANRIL. Then, the relative expression of ANRIL was calculated by the 2^-ΔΔCT^ value method, and GAPDH was used as a housekeeping gene. The specific primers for ANRIL, ATR and GAPDH used for RT-PCR are listed in Table S2. All the primers were all synthesized by Sangon (Shanghai, China). For determining the half-life of ARNIL, H1299 cells were pretreated with Actinomycin D and the level of ANRIL was measured at different time [8]. For ATM, ATR, and DNA-PKcs inhibitors, cells were pretreated with ATR inhibitor (VE821), ATM inhibitor (Ku55933) and DNA-PKcs inhibitor (Nu7441) for 1h, after cells were irradiated and then ANRIL was determined. Each PCR amplification was performed in triplicate to verify the results.

**Western blotting**

Cells in the logarithmic growth phase were placed in a 60 mm dish at the appropriate density and cultured in an incubator. Proteins were extracted from irradiated cells by using M-PER Mammalian Protein Extraction Reagent (Thermo Fisher Scientific, Taiwan, China) according to the manufacturer's instructions. Equal amounts of proteins were separated on a 10% sodium dodecyl sulfate-polyacrylamide gel electrophoresis gel (Beyotime, Shanghai, China) and transferred to nitrocellulose membranes (Millipore, USA). Five percent skimmed milk was used to block the membranes for 1 hour, and then, the membranes were probed overnight at 4°C with the primary antibodies listed in Table S3. Next, the membranes were incubated with specific secondary antibodies (Servicebio, Wuhan, China) for 2 hours at room temperature. Peroxidase labeling was visualized via enhanced chemiluminescence labeling using an ECL Western blotting detection system (Thermo Fisher Scientific, Waltham).

**Comet assay**

The comet assay was used to detect DNA double-strand breaks. First, we prepared glass slides by immersing them in molten 1% normal melting agarose, cleaning the upper side immediately, and maintaining the low melting agarose (LMA) at 40°C in a water bath. Second, a neutral lysis solution (58.44 g NaCl, 5.584 g Na_2_EDTA, and 0.61 g Tris in 500 ml double distilled water, pH 8.2-8.5) and TBE buffer (0.744 g Na_2_EDTA, 5.564 g boric acid, and 10.902 g Tris in 500 ml double distilled water, pH 8.2-8.5) were prepared for subsequent lysis and electrophoresis. Third, the single-cell suspension was mixed with ice-cold Ca^2+^-free and Mg^2+^-free PBS to a final volume of 2×10^4^ cells/ml, and then, 0.4 ml of the suspension was dipped in 1.2 ml LMA. Finally, 0.5 ml of the single-cell suspension was pipetted and immediately tiled on the surface of the precoated slide. When the suspension solidified, the gels were lysed in neutral lysis buffer (Triton X-100 was added to a final concentration of 1% before use) overnight in the dark. After a gentle rinse with TBE, the slides were electrophoresed at 4°C for 25 minutes at 7 mA and 25 V in TBE. Then, the gels were dyed with PI (10 μg/ml) for 20 minutes and rinsed with double distilled water. Finally, all the gels were observed by fluorescence microscopy, and over 100 comet images of each slide were analyzed to determine the tail DNA % and tail moment using CASP 1.2.3b2 (CASPlab, Wroclaw, Poland).

**Immunofluorescence staining**

Cells plated on 22X22 mm^2^ cover slips in 6-well plates were irradiated and fixed in 4% paraformaldehyde for 30 minutes at room temperature, permeabilized in 0.5% Triton X-100 buffer and then blocked in 1% BSA for 1 hour at room temperature. Then, the cells were incubated with a γH2AX (Ser139) monoclonal antibody (Abcam), 53BP1 antibody (Abcam), RPA2 antibody (Abcam) and ATR (Abcam) antibody at 4°C overnight and washed twice with PBS. Subsequently, the cells were incubated with a FITC-labeled anti-mouse antibody (Abcam) and a Texas Red-labeled anti-rabbit antibody (Abcam) at room temperature for 2 hours. DNA was stained with 2 g/mL DAPI for 15 minutes in the dark. Images were obtained under a [confocal](javascript:;) [microscope](javascript:;) (BRUKER, USA) with the NIS-Elements Viewer 4.20 capture system. We observed 3 slices of each specimen, and 5 high-power visual fields were randomly selected from each slice to count the number of foci or positive cells by ImageJ software. Then, the percentage of positive cells was calculated as an average.

**Animal experiments**

Four-week-old male BALB/c-nu/nu nude mice (obtained from the Experimental Animal Center of Naval Medical University) were kept in the Animal Room of the Department of Radiation Medicine, Naval Medical University. Nude mice were kept in a constant temperature environment at 22-25°C, alternating between day and night for 12 hours, with sufficient food and autoclaved drinking water. NC and ANRIL-KD H1299 cells and the vector or ANRIL-OE H460 cells were collected and injected subcutaneously into both thighs of mice at a concentration of 5×10^6^. All the cells were in the logarithmic growth phase, and the mice were anesthetized in an isoflurane animal anesthesia apparatus. For tumors derived from ANRIL-OE H460 cells, 10 nude mice were treated for tumors and continued to be raised in the animal room until the tumors were visible (approximately one week, 100 mm^3^). The 10 nude mice that developed tumors were divided into the nonirradiation group and irradiation group according to the principle of random distribution, with 5 mice in each group. After irradiation, the two groups of nude mice were maintained for 2 weeks. Then, the mice were sacrificed, the tumors were excised, and the weight and volume of the tumors were measured. Tumor volume (mm^3^) = (long diameter×transverse diameter^2^)/2. For tumors derived from ANRIL-KD cells, nude mice with tumors were randomly divided into 4 groups. After exposure to local irradiation, 3 mice were sacrificed at 0, 8, and 24 h to detect the activation of HR repair factors. 3 mice were used to establish the growth curve and were sacrificed 2 weeks after irradiation. The overall design of the tumor experiments is shown in Fig. S5A. A total of 3 parallel experiments were performed in this experiment. The animal’s studies were approved by the Animal Research Ethics Committee of Naval Medical University and were conducted in accordance with the relevant guidelines and regulations.

**Immunohistochemical (IHC) and TUNEL staining**

When tumors derived from H1299 NC and ANRIL-knockdown H1299 cells, as well as the H460 vector and ANRIL-overexpressing H460 cells, reached a volume of 100 mm^3^, mice were subjected to 10 Gy local irradiation, and the tumors were isolated 8 or 24 hours after irradiation for immunohistochemistry. The tumors were fixed in 4% paraformaldehyde for 24 hours, embedded in paraffin, and cut into 3-μm sections. The sections underwent dewaxing, rehydration, antigen retrieval, and blocking and were then incubated with antibodies against RAD51, γH2AX, ATR, Ki67, and RPA2 overnight at 4°C and washed with PBS 3 times. The sections were then incubated with an HRP-conjugated secondary antibody for 1 h at room temperature, washed with PBS 3 times, and stained with DAB and hematoxylin. Finally, the sections were dehydrated with different concentrations of ethanol and mounted with coverslips. The slides were used for TdT-mediated dUTP nick-end labeling (TUNEL) according to the manufacturer's instructions for the TUNEL kit (Roche, Switzerland). The sections were observed under a [microscope](javascript:;) (Nikon-ETi, Japan) equipped with a NIS-Elements Viewer 4.20 capture system. Three slices of each specimen were observed, and 5 high-power visual fields were randomly selected from each slice. ImageJ software was used to analyze the positive cell percentage. The IHC Profiler plug-in was used to automatically score the staining of the sample, and then, the Trainable Weka Segmentation plug-in was used to count positive and negative cells.

**RNA immunoprecipitation (RIP)**

Cells were used to perform RNA immunoprecipitation (RIP) experiments using the Magna RIP™ RNA-Binding Protein Immunoprecipitation Kit (Millipore, Bedford, MA) according to the manufacturer’s instructions. Cells under good growth conditions were spread in a 10 cm dish at the appropriate density and were irradiated after they had grown to the appropriate amount (cell number in each group> 2×10^7^). Then, the cells were rinsed with PBS and centrifuged at 1500 rpm for 5 minutes at 4°C, and the supernatant was discarded. Next, the cells were resuspended in 100 μL of RIP lysis buffer and pipetted until homogeneous on ice for 5 minutes. Magnetic beads were washed with RIP wash buffer and incubated with 5 μg of the anti-IgG antibody (Millipore, Bedford, MA), anti-ATR antibody (Cell Signaling Technology, Danvers, USA), anti-RPA2 (Cell Signaling Technology, Danvers, USA) or anti-RAD51 (Cell Signaling Technology, Danvers, USA) for 30 minutes at room temperature in 100 μL of RIP wash buffer. For RIP experiments for ATR fragments, Flag wild type (WT, a kind gift from prof. Qiang Liu, Institute of Radiation Medicine, Chinese Academy of Medical Science), Flag-ATR-N (Addgene, # 53767) and Flag-ATR-C (Addgene, #53769) were used and immunoprecipitated with Flag primary antibody (Sigma). After a brief centrifugation, the supernatant was discarded, and the unbound protein antibodies on the magnetic beads were washed away with RIP wash buffer. Then, we centrifuged the cell lysate at 4°C and 14000 rpm for 10 minutes and collected 100 μL of the supernatant. The supernatant was incubated with 900 μL of IP buffer containing magnetic beads conjugated with different antibodies at 4°C overnight. Before incubation, 10 μL of the sample buffer was removed and marked as the input for later Western blot experiments. The sample buffer was then washed with RIP wash buffer and was used in subsequent Western blot experiments for verification after heat denaturation. At the same time, 150 μL of Proteinase K buffer was added to the sample buffer to dissolve protein. Then, immunoprecipitated RNA was isolated, and coprecipitated RNAs were detected by qRT-PCR.

**RNA pulldown assay**

The cDNA sequence of ANRIL and different fragments were cloned into pBluescript II SK(+). Biotin-labeled RNAs were transcribed *in vitro* using a biotin-labeling mix and T7 polymerase in the linearized pBluescript II SK plasmid following the manufacturer’s instructions (Large Scale RNA ProductionSystem-T7, Promega). For the RNA pulldown assay, cells were treated with the RNA 3'-End Desthiobiotinylation Kit and Pierce™ Magnetic RNA-Protein Pull-Down Kit (Thermo Fisher, USA). Cells were rinsed with PBS and then resuspended in 1 ml ice-cold PBS. Then, we centrifuged the suspension at 4°C and 1200 rpm for 3 minutes. Next, the cell pellet was suspended in 400 μL of dilution buffer (with a protease inhibitor cocktail) and centrifuged at 4°C and 12000 rpm for 10 minutes. The cell supernatant was collected for use in the next experiment. Pierce nucleic acid compatible streptavidin magnetic beads were washed twice with wash buffer to remove the stock solution and were resuspended in RNA capture buffer. We added labeled biotin-ANRIL to the beads and incubated them for 15-30 minutes. Then, the beads were washed twice with wash buffer and resuspended in Protein-RNA Binding Buffer. Next, we constructed the RNA pulldown reaction system according to the manufacturer’s instructions and incubated the beads at 4°C for 1 hour in a rotary shaker. Finally, we eluted the proteins with 50 µL biotin elution buffer after washing the beads with wash buffer and detected the proteins by SDS-PAGE and mass spectrometry analysis.

**RNA sequencing**

Total RNA was isolated from ANRIL NC and ANRIL-KD H1299 cells, and the RNA quality was determined using a nucleic acid quantitative detector (GeneQuant Pro, USA). One microgram of RNA was used for sequencing. The cDNA library was prepared according to the manufacturer’s protocol and was then sequenced on the Illumina HiSeq X-ten platform at ShBio Biotech (Shanghai, China). The quality of the raw reads was determined using the FastQC tool kit. After RNA sequencing, the raw reads were preprocessed by filtering our rRNA, short-fragment reads and other low quality reads. Then the clean reads were mapped to human GRCh38 reference genome with Hisat2 software (Version 2.0.4). Afterwards, Stringtie (version:1.3.0) was run with a reference annotation to generate FPKM values for known gene models. Differentially expressed genes were identified using edgeR software. The average expression abundance of a DEG (differentially expressed gene) in at least one sample was more than 1 fragment per kilobase million, with a false discovery rate (FDR) < 0.01 and abs (fold change) > 2.

**RNA FISH**

A fluorescence in situ hybridization (FISH) assay was conducted to investigate the distribution of ANRIL in H1299 cells by using a FISH Kit (RiboBio, China) according to the manufacturer’s instructions, and the Cy3-labeled ANRIL probe was obtained from RiboBio. RNA FISH experiments were performed on the H1299 cells with a FISH Kit (RIBOBIO, China) according to the manufacturer’s instructions. The H1299 cells were plated on a 22×22 mm^2^ cover glass in 6-well plates and divided into the internal reference U6 group and ANRIL group. When the density of the cells reached approximately 70-80%, the cells were washed with PBS and fixed with polymethyl methacrylate for 10 minutes. After addition of the prehybridization solution, the U6 probe-containing hybridization solution was added to the cells in the U6 group, and the ANRIL probe-containing hybridization solution was added to the cells in ANRIL group overnight at 37°C in the dark. After labeling with the probe, the cells were stained with DAPI for 10 minutes, and images were taken under an immunofluorescence confocal microscope (BRUKER, USA).

**RNA secondary structure prediction**

The secondary structure of ANRIL was predicted by online software (the RNA fold web server: http://rna.tbi.univie.ac.at/cgi-bin/RNAWebSuite/RNAfold.cgi). The ANRIL sequence was uploaded onto the server, and the secondary structure of ANRIL calculated based on the recommended settings and parameters. For fold algorithms and basic options, “minimum free energy and partition function” and “avoid isolated base pairs” were chosen.

**Immunoprecipitation**

Eight hours after ANRIL NC and ANRIL-knockdown cells were irradiated at a dose of 8 Gy, proteins were extracted. Then, proteins were immunoprecipitated with an ATR-specific antibody using the Pierce™ Co-Immunoprecipitation Kit (Pierce, #26149) according to the manufacturer’s instructions. After immunoprecipitation, proteins were subjected to SDS PAGE and immunoblotted with an antibody against ubiquitin. Degradation of the ATR protein was confirmed by using a proteomic inhibitor, MG132.

**Irradiation**

γ-Radiation was performed using a ^60^Co source at the Radiation Center (Faculty of Naval Medicine, Naval Military Medical University, China). Cells were irradiated with different doses of radiation as indicated. The dose rate was 1 Gy/min. Mice received a pelvic cavity local ^60^Co 10 Gy exposure, with the upper region of the body shielded with lead.

**Statistical analysis**

All experiments were performed with at least three independent experiments. In general, Student’s two-tailed unpaired t test was used to compare differences between two groups. One-way analysis of variance followed by the Newman–Keuls multiple comparison test were used to compare more than two groups. All data are expressed as the means±standard deviation (SD) for each experiment. A P value of <0.05 was considered to indicate a statistically significant result. GraphPad Prism 6 Software (GraphPad Software Inc., La Jolla, CA) was utilized for all statistical analyses and construction of graphs.
