# Supplementary material for: Long non-coding RNA ANRIL promotes homologous recombination-mediated DNA repair by maintaining ATR protein stability to enhance cancer resistance
Source: Mol Cancer. 2021 Jul 5;20:94. doi: 10.1186/s12943-021-01382-y (PMC8256557; doi:10.1186/s12943-021-01382-y)
Supplement: Supplementary file 9 — Additional file 9. [file 12943_2021_1382_MOESM9_ESM.docx]

**ANRIL knockdown vector construction:**

Carrier name：pleno-gph

Component order：CMV-MCS-EF1α-GFP-t2a-PURO

Vector map：


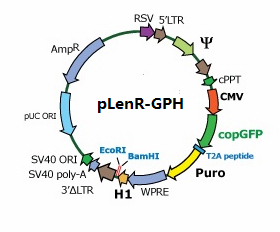


**ANRIL overexpressing plasmid construction:**

Carrier name：pLenO-GTP

Component order：CMV-MCS-EF1α- GFP-Puro

Vector map：

| **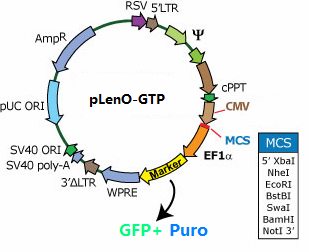** |
| --- |

**ANRIL overexpressing sequence:**

agctacatccgtcacctgacacggccctaccaggaacagccgcgctcccgcggattctggtgctgctcgcgtccccgctcccctattccccttattttattcctggctcccctcgtcgaaagtcttccattcttcaaactagattatttaaaaatgaaaaaggaagaaaggaaagcgaggtcatctcattgctctatccgccaatcaggaggctgaatgtcagttttgaactaaaagccgctccgctcctcttctagatttggaaaacaagcgaaattaaactaaaccgctgcacgcctctgacgcgacatctggacacggcgcggcgctggcgctgccggagctgtcgacccggcctggcgccggactaggactatttgccacgacatttcaaaggattccaagagagaatattggtgtccatgctgtgatgattcctcagctcctctcatctgatctccgtcctggcccccatgactttctttgtggtagttagggtgtggtatgtgccactgaggcccacacctattgctgcaatttatagcactgatctgtcatcaataccacttgctgtcttggatgtgaagatgatttttcctgcagggattccctctacaaaattaaaaacactgggcatgtggaaataatattcatgctttaaattgtcttttctcttcactacaccaggggtccccaacccctaggccacagactgtggccctagtgtagtgaatagaaaagacaatttaaagcgtgaatattatttcctcatgcccagtgtttttaattttgtactggtctgtggcttgttagaaaccaggctgcacagcagaaggtgggcagcagatattgagaaaccacagaaagagagaagttaaataattttcctgtcaaagaccacatcaatgatgaagccagaatttgagctcatgtacttaaccactggactacctgcctgccctgtcgaggaacagctaagtgtcccttttgatgagaagaataagcctcattctgattcaacagcagagatcaaagaaaagacttctgttttctggccaccagatatatgttatctgtgcttaaagaattgaaaaacacacatcaaaggagaattttcttggaaagagagggttcaagcatcactgttaggtgtgctggaatcctttcccgagtcagtactgctttctagaagaaaaccggggagatctatttggaatgtatctaactccaaagaaaccatcagaggtaacagtagagacggggtttcaccatgttggccagactggtcttgaactctcgacctcgtgattcgcccgcctcggcctcccaaagtgctgggattacaggtgtgagacaccacacccggcggatagagagaattttgacagtctctccaatgaacgccttcactgatatccaaagcatgaaggacacaccagggaaaaacatagacctaacacaggacaaatggaattattagaaacattttctagcagaagaacactattctgttgccatttgaatctttgcttctttctaggtttgacaatgagcctatcatataagcccaaatgtaaacagaaagaggttgaatcagtcacgataagcccaattatgctgtggtaacaaacaacctcaaaatctcattggcttaaaatatacagaattattcttactcatggcacatatccatctatcatctgcaggggatctgctcactgaagtcacttaggaacttggactgatggaacggccactttttggtcactatatgtattaatctgttttaatcctgctgataaagacccaaaattgggaacaaaaagaagtttaactagacttacagttccgcatggctgaggaggcctcagaatcatggtgggaggcgaaaggcacttcttacatggtggcagcaagagaaaaatgaggaagaagcaaaagcggaaacctctgataaacccatcagatcttatgagacttattccactatcaagagaatagcatgggaaagactggctcccataatttacctccctctgggtccctccctcaacatgtgggaattctgggagaaacaattcaagatatgacacattcataatttaaacagaagcctacgaagaactcataaattaaaagaagataatcttttcacaaggtgatggaggctttttattttgccacaaaaccactggtgacgttgcctgtggccaccttggagaagacactggaggcctgggacatggagactgcttttctgcagaaaccacatcccttggagtaatgagctacacctacctcaattattcagtgcagtacaacactccagacagggtctcactctgtcacccaggctggagtgtattggcatgattacagctcactgcaaccttgaactcccaggctcaaacctgagcagctgggactacagatgcaccaccatgcatggtaccagagatataataatgagaaacagacatgctccctcccctcattgaggttacagcttagtgtggagacacacagatgcctaacgcactatggtatggaaggtgctatggacacagtgctcaaatccatgatctacataggtggagaacttcagtagaggaagtggcaggaatttgggaatgaggagcacagtgattaaactggggccattcatatgagagtttaagaactcagaccagtgacttagattggcttctctcacatggcaagaaacattgctgctagcacttcccgagttctacgttctacaacatccaccactggatcttaacatagacgtaagatcaaatgcaatagcatgtcaaacaatgtgtaactccagttatacaaacattactgtatctcattggggatacgaagctctacacacttgaagatggtgaaggaatataaaaatctatgtctcacagtccagacttggagtacaagtaataagaagaataaaacttaatcccttaagtagattcaccataagttagctcagagcaattccagtgcaagtatggtctgtgatccagtagtatcttacagacagcaagttgaacattgtgggatgcatgagctattgaggcctttgcagctttctgctacatggaggctagggccagagtcaagatttatgctttgcagcacactggtcagctgtttttgcaaatcagattaaatgatttttaaatgaggctgagagcatgggagatactaatgtgtgtttccttgtgagctactgcataagttaggaaattgaaatacagaaagatgaaaagtgatttgcccaagcatatagatcaaagctgtggcagaaccaggactggaacctatatctctctactaatggtttttttaaaaaaataaccttgtttcaaaaatattaaaaagtcacaagaaaggtaaacatgtggataaacaaaatgaagaaaataaaaattatccagtaataacatattggcatatgtctttctggtatattttcctgtgttgtcatcattatcatctccatcatcattatatccatcattatcatcatcatcatcatcatcatcatcattatcatcaccatagtgaacatgtaatgcttacctagtgccagatgctgtctaggcattttacatgtgttactggtaactcatgtaatcctcataacaaccttataaggtggttgctattatccccatgttacatatgaagagacagaagcataaagaagttgcaccgctggtaattggctgggatttgaacttaagcagtctaaccttagagtaatgattttaacaactatgctatatacatacaaatttacaaaataaaactgggctcagacaataaaaaaaaaaaaaaaaaaaaaaaaa
